# Alteration of the Systemic and Microcirculation by a Single Oral Dose of Flavan-3-Ols

**DOI:** 10.1371/journal.pone.0094853

**Published:** 2014-04-16

**Authors:** Kodai Ingawa, Nozomi Aruga, Yusuke Matsumura, Masahiro Shibata, Naomi Osakabe

**Affiliations:** Department of Bio-Science and Engineering, Shibaura Institute of Technology, Fukasaku, Munumaku Saitama, Japan; University of Catanzaro Magna Graecia, Italy

## Abstract

Several systematic reviews have reported that flow mediated dilatation (FMD) was significantly increased in subjects after ingestion of chocolate that contains flavan-3-ols; however, the mechanisms responsible for this effect are not clear. In this study, we evaluated the effects of a single oral dose of flavan-3-ols on the systemic circulation and microcirculation in the cremaster muscle using intravital video microscopy *in vivo*. The cremaster muscle in rats was spread over a plastic chamber and a gastric tube was placed into the stomach. Blood flow in the cremasteric artery was determined using a laser Doppler flowmeter, while blood pressure and heart rate were measured by the tail-cuff method. Red blood cell velocity in arterioles and blood flow in the artery were significantly increased 5 min after the administration of 10 mg/kg flavan-3-ols compared with distilled water treatment. The number of capillaries recruited in the cremaster muscle was also significantly increased 15 min after treatment. Microscopic observation confirmed that increased shear stress on endothelial cells was maintained during the measurement period. The mean arterial blood pressure and heart rate were also significantly elevated soon after administration and returned to baseline before the end of the observation period. Plasma nitrate and nitrite levels, and NO phosphorylation of aortic tissue were significantly increased at 60 min after administration of flavan-3-ols. According to these results, a single oral dose of flavan-3-ols elevates blood pressure and flow transiently, and these effects induce NO production through increased shear stress on endothelial cells.

## Introduction

Cocoa bean, the seed of *Theobroma cocoa*, is one of the ingredients in chocolate and cocoa. It is known to be rich in polyphenols, such as the flavan 3-ol monomers, (+)-catechin and (-)-epicatechin; and oligomers, such as the B-type flavan 3-ols linked by C4-C8 bonds [1]–[3]. Recent epidemiological meta-analyses have suggested that ingestion of chocolate reduces the risk of cardiovascular disease and stroke [4], [5]. Numerous studies support the idea that flavan-3-ols in cocoa reduce the risk of cardiovascular disease by improving hypertension, dyslipidemia and glucose intolerance [6]–[12]. In addition, flow-meditated dilation (FMD), which is a non-invasive method to assess endothelial function, was shown to be significantly increased shortly after ingestion of dark chocolate in healthy or mild hypertensive subjects [13]–[15]. It is well known that endothelial function is affected by cardiovascular risk factors, and can also be influenced by exercise [16], [17] or behavioral factors [18]. It was reported that the blood NO metabolite level was increased after ingestion of chocolate; however, there is little information about the mechanisms responsible for this effect.

In this study, we evaluated the acute effects of flavan-3-ols on the systemic circulation and microcirculation in skeletal muscle using intravital video microscopy under physiological conditions. We also examined changes in eNOS phosphorylation in aortic tissue and nitrate/nitrite levels in blood to elucidate the mechanisms of the acute response to flavan-3-ols.

## Materials and Methods

### Materials

Urethane and Krebs- Ringer bicarbonate buffer were purchased from Sigma Chemicals (St. Louis, MO, USA). The flavan-3-ol fraction was provided by Meiji Co., Ltd (Tokyo, Japan), and the concentrations of polyphenols are shown in Table 1. The total polyphenol content was determined by the Prussian blue method [19], and each polyphenol was measured by high performance liquid chromatography (HPLC) [20].

**Table 1:** Concentration of polyphenols in flavan-3-ol fraction.

Concentration (%)	
Total polyphenol^1^	72.37
(+)-catechin^2^	4.56
(−)-epicatechin^2^	6.43
procyanidin B2^2^	3.93
procyanidin C1^2^	2.36
cinnamtannin A2^2^	1.45

1 Total polyphenols were determined by the Prussian blue method using (-)-epicatechin as the standard.

2 Each flavan-3-ol concentration was determined by HPLC.

### Animals and diets

This study was approved by the Animal Care and Use Committee of the Shibaura Institute of Technology (Permit Number: 27-2956). All animals received humane care under the guidelines of this institution. Male Wistar rats weighting 200–250 g were obtained from Saitama Experimental Animal Supply (Tokyo, Japan). The rats were kept in a room with controlled lighting (12 h light and dark cycles at a regulated temperature of 23–25°C). The diet was certified diet obtained from the Oriental Yeast Co., Ltd., Tokyo, Japan.

### Experimental procedures

Thirty two animals were fed a basal diet for 4 days and then allocated to two groups, with each group treated 4 ml/kg distilled water (vehicle group; n = 16) or 10 mg/kg flavan-3-ol fraction (flavan-3-ols group; n = 16), flavan-3-ols was dissolved in distilled water. Microscopic observation was carried out using eight rats then sacrificed under anesthesia, laser Doppler and tail cuff measurement was performed the other eight rats in each group. A gastric tube was inserted under urethan anesthesia (1 g/kg, SC). The cremaster muscle was exteriorized and carefully spread out on a dedicated plastic chamber with an optical port for transillumination; the surface was superfused with Krebs-Ringer bicarbonate buffer (pH 7.3–7.4) in an environment with 95% N_2_ and 5% CO_2_ at 37°C. Thirty min after post-surgical equilibration period, a single unbranched arteriole with a resting inner diameter of 15–20 µm was selected from the microscopic images for measurement of red blood cell velocity and diameter of the vessels. After 15 min of baseline observation, 10 mg/kg of flavan- 3-ols or distilled water was administered orally to animals through the fixed gastric tube. The dosage of flavan-3-ols was determined according to a preliminary experiment (data not shown). The microcirculation was visualized by placing the chamber on a three-way movable stage, and the cremaster was transilluminated with a 150-W halogen light. The microcirculation was observed using an intravital microscope (M5A, Olympus) equipped with a charge- coupled video camera (DXC-107S, Sony, Tokyo). The images were displayed on a high-resolution television monitor at a final magnification of 1450× and stored for off-line analysis. Red blood cell (RBC) velocity and the number of newly-recruited capillaries were measured using a video image with 8-bit gray levels at a resolution of 512×512 pixels. RBC velocity was measured by monitoring the change in the position of a RBC over time in successive frames, and each velocity measurement was repeated 3 times. The number of newly-recruited capillaries was counted over an 846×307 µm area of the cremaster muscle throughout the observation period.

**Figure 1:**
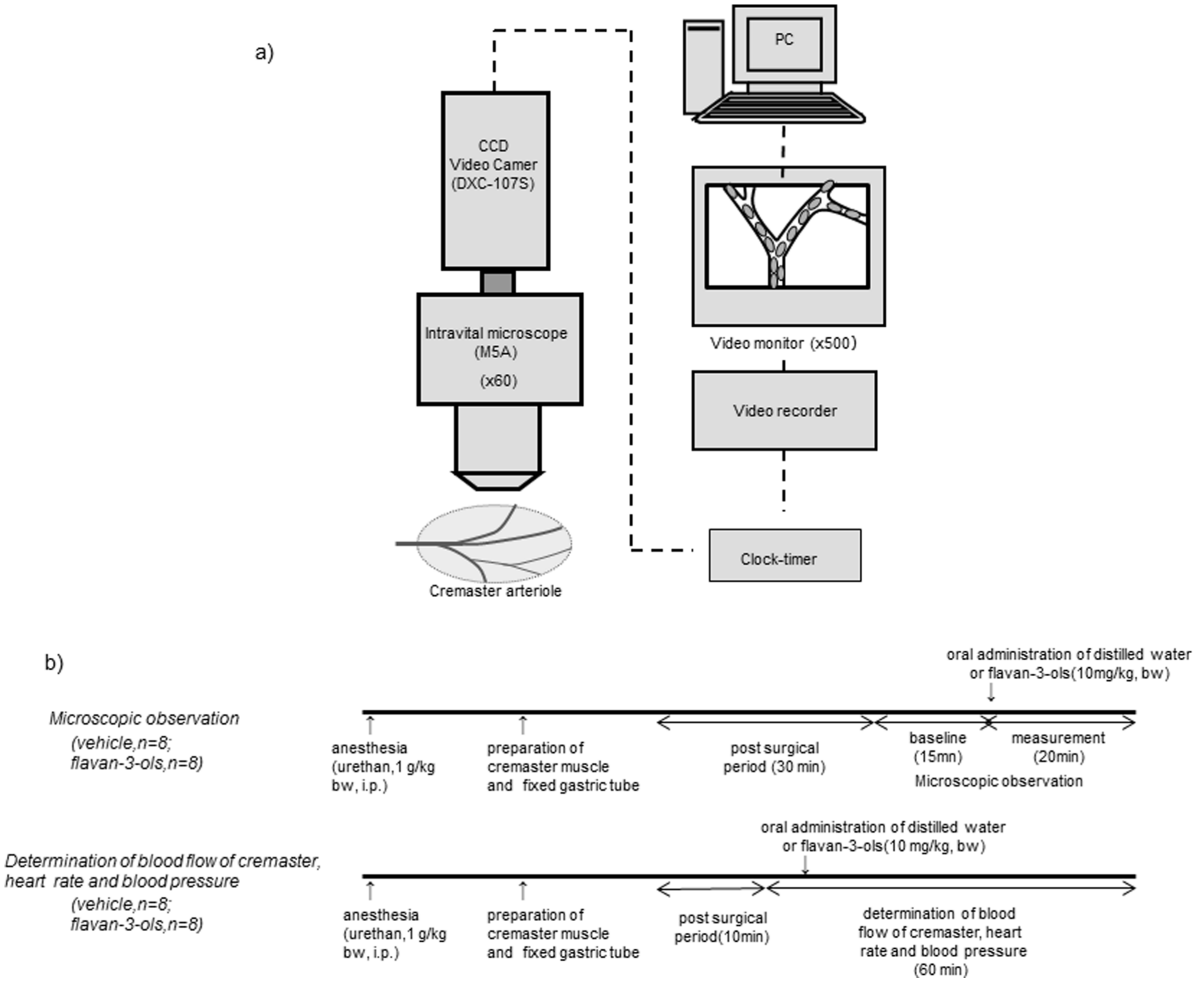
Schematic diagram of the intravital microscopic analysis system (a), and time line of the present experiment (b).

A schematic diagram of the experimental apparatus is shown in Fig.1 (a). The cremasteric arterial blood flow was determined using a laser Doppler blood flowmeter (Periscan PIM-2, Perimed Co. Ltd.), and blood pressure and heart rate were determined simultaneously by the tail-cuff method (BP-98A Softron, Tokyo Japan) every 6 min. The cremaster muscle was exteriorized under anesthesia as described above. After all the measurements were completed, 4 to 5 ml of blood sample was collected from the abdominal vein using a heparinized syringe. The aorta was removed by dissection, snap frozen in liquid nitrogen and stored at -80°C until analysis.

### Western blotting analysis

Aortic tissue was homogenized in a microtube with lysis buffer (CelLytic™ MT cell lysis reagent; Sigma Aldrich, Japan) containing a protease inhibitor (Sigma Aldrich, Japan) and 0.2% SDS. Protein concentration was measured by the Bradford method. Protein (50 µg) was separated by SDS-PAGE using a 4–12% Bis-Tris gel and transferred onto a polyvinylidene difluoride membrane (Life Technology). The membrane was blocked with membrane-blocking reagent (GE Healthcare) for 1 hour. After blocking, the membrane was probed with a rabbit polyclonal primary antibody against phosphorylated eNOS (Ser 1177, 1∶800; sc-12972 Santa Cruz Biotech, Dallas, TX, USA) and an α-tublin antibody (1∶2000; ab4074, Abcam) for 2 hours. After the primary antibody reaction, the membrane was incubated with appropriate horseradish peroxidase-conjugated secondary antibodies (1∶100000) for 1 hour. Immunoreactivity was detected by chemiluminescence using the ECL Select™ Western Blotting Reagent (GE Healthcare). Fluorescence band images were analyzed using Just TLC (SWEDAY) analysis software. Values were normalized to those for α-tubulin.

### Measurement of nitrate and nitrate in plasma

The plasma nitrate and nitrite concentrations were determined by using the Griess reaction [21]. Briefly, plasma was incubated with the same volume of nitrate reductase in 0.1 M potassium phosphate buffer containing 1 mM βnicotinamide adenine dinucleotide phosphate (NADPH) and 2 units of nitrate reductase/mL. Samples were allowed to incubate overnight at 37°C. Griess reagent (1% sulphanilamide, 0.1% naphthyl- ethylenediamine dihydrochloride in 5% phosphoric acid) was added, and the samples were incubated for an additional 115 min at room temperature. The total amount of nitrite was measured at 540 nm.

### Data analysis and statistical methods

Data are expressed as the means and standard deviations. Statistical analyses were performed using Student's t-test or Dunnett's test. P<0.05 was considered significant.

## Results

### Changes in microcirculation in the cremaster muscle after treatment with flavan-3-ols

**Figure 2:**
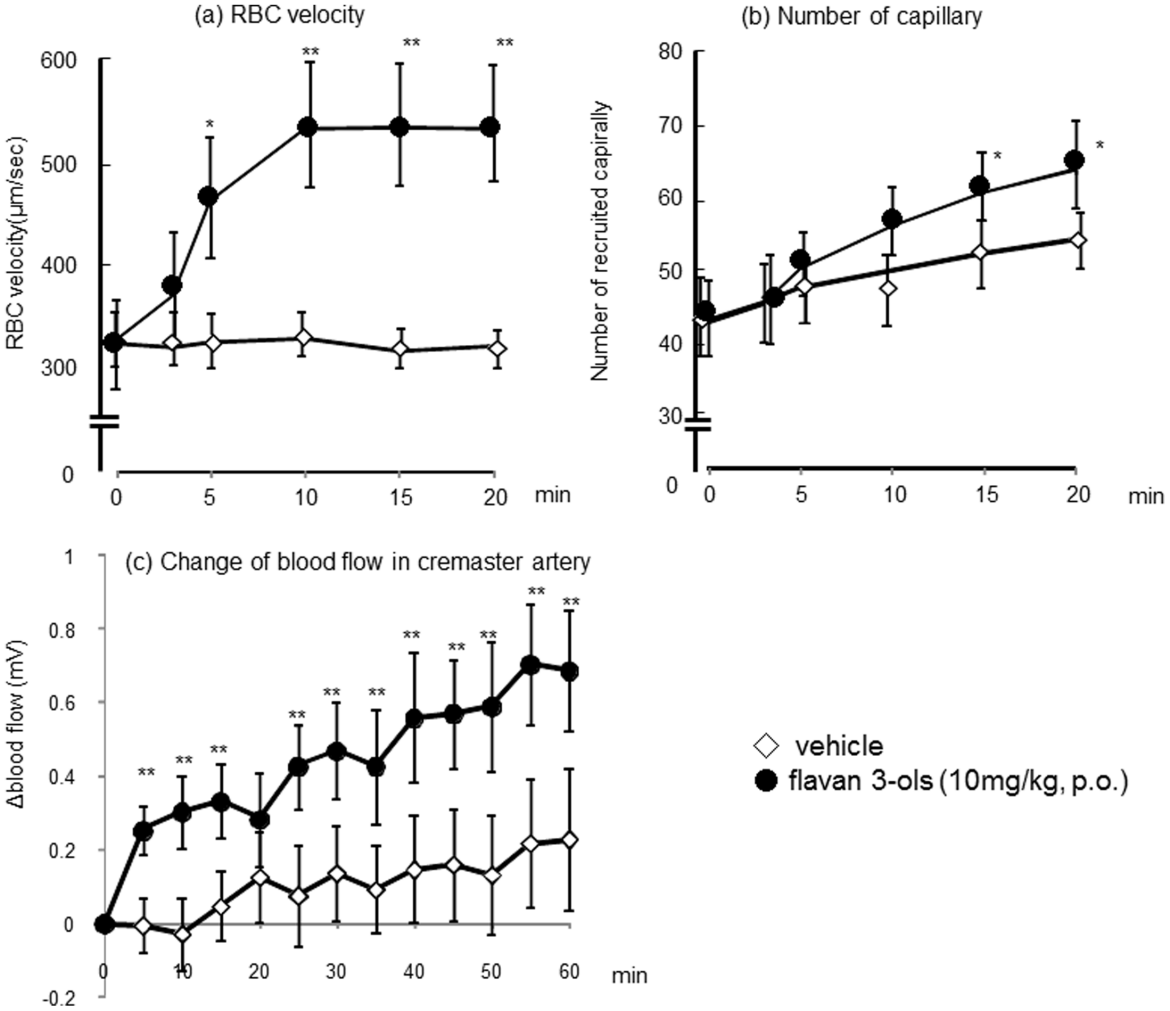
Acute effects of a single dose of flavan-3-ols on several microcirculatory parameters in the cremaster muscle: RBC velocity in the arteriole (a), the number of capillaries recruited (b), and blood flow in the cremasteric artery (c). Each value represents the mean and standard deviation (n = 8). *p<0.05 and **p<0.01 compared with vehicle-treated control rats.

Changes in the microcirculation of the cremaster muscle before and after oral administration of flavan-3-ols are shown in supplemental videos 1 (before treatment) and 2 (5 min after flavan-3-ols treatment). The marked elevation of RBC velocity in the arterioles can be seen in the video image, and this confirms that endothelial cells were exposed to severe shear stress. The average RBC velocity in the arterioles shortly after treatment with flavan-3-ols is shown in Fig.2 (a), and a significant difference was observed compared with vehicle from 5 min after treatment until the end of the observation period. At the end of observation period, RBC velocity in flavan-3-ols group was increased about 1.7-fold compared with vehicle group. The number of capillaries recruited in the rat cremaster muscle is shown in Fig. 2(b). A significant increase in capillary recruitment was observed after flavan-3-ols treatment compared with vehicle treatment at 15 and 20 min (1.25 and 1.35-fold increase) after administration. Blood flow in the cremasteric artery is shown in Fig 2 (C). There was a significant difference between vehicle-treated and flavan-3-ols-treated rats 5 min after treatment, and the elevated blood flow in the flavan-3-ols group was maintained during the measurement period. At the end of measurement period, blood flow of cremaster artery in flavan 3-ols group was increased 3.5-fold compared with vehicle group.

### Changes in heart rate and blood pressure

**Figure 3:**
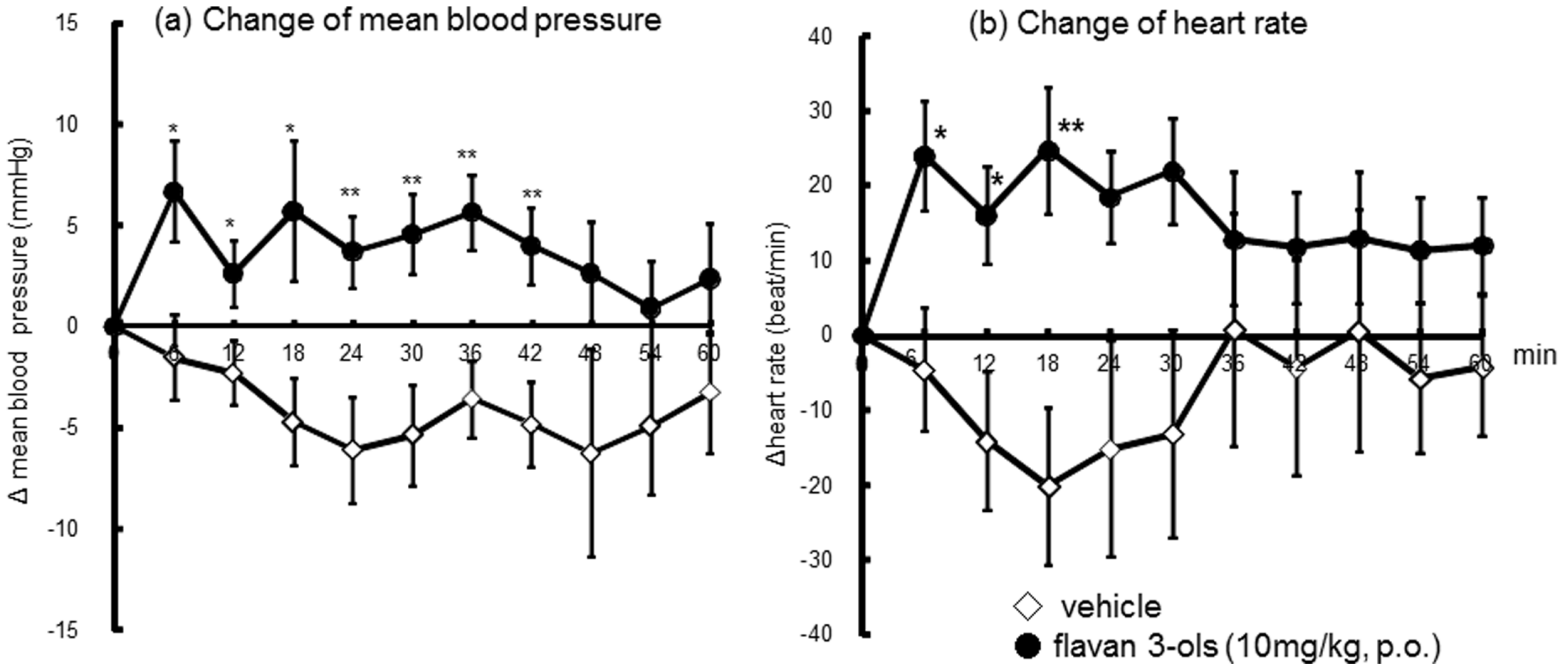
Acute effects of a single dose of flavan-3-ols on changes of mean blood pressure (a) and heart rate (b) in rats. Each value represents the mean and standard deviation (n = 8). *p<0.05 and **p<0.01 compared with vehicle-treated control rats.

Changes of blood pressure and heart rate measured by the tail cuff method are shown in Fig. 3 (a,b). Mean blood pressure was significantly increased soon after treatment with flavan-3-ols;the elevation from 4 to 7 mmHg was maintained until 42 min and then returned to baseline (Fig. 3a). A similar result was observed for heart rate: there was a significant increase (from 18 to 25 beat/min) until 18 min after administration of flavan-3-ols and then a return to baseline.

### eNOS phosphorylation in aorta and nitrate and nitrite levels in blood

**Figure 4:**
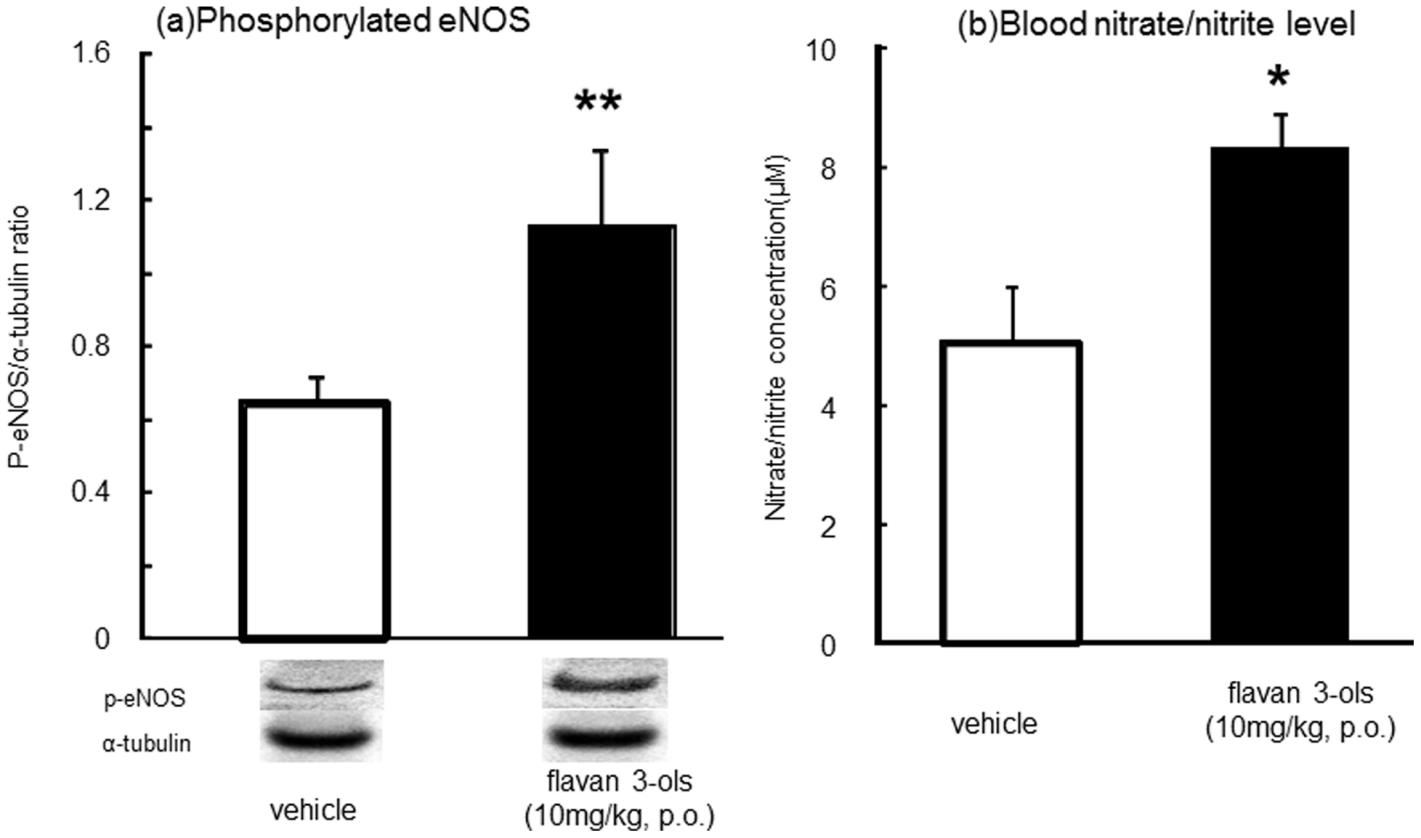
eNOS phosphorylation and blood nitrate/nitrite levels 60 min after administration of flavan-3-ols. Each value represents the mean and standard deviation (n = 8). *p<0.05 and **p<0.01 compared with vehicle-treated controls.

Fig.4 shows eNOS phosphorylation in the aorta (a) and blood nitrate and nitrite levels (b) 60 min after administration of flavan-3-ols. Aortic phosphorylated eNOS was nearly doubled compared with vehicle group, and blood nitrite and nitrate concentrations were also significantly elevated (1.7 fold) by treatment with flavan-3-ols.

## Discussion

In the present study, we observed increased RBC velocity in the cremasteric arterioles (Fig.2a) and blood flow in the cremasteric artery (Fig.2b) soon after oral administration of flavan-3-ols, and these observations indicate severe shear stress on endothelial cells (video 1 and 2). We also found that mean blood pressure and heart rate were significantly increased immediately by the ingestion of flavan-3-ols. The significant elevation of blood pressure was maintained 42 min after treatment with flavan-3-ols and then returned to baseline (Fig.3). The heart rate was also significantly increased during the 18-min measurement period and then also returned to baseline. As the changes of heart rate and blood pressure were shown in Fig.2, there was slight reduction of heart rate and blood pressure in vehicle group during the early part of measurements. Though there were no significant changes in absolute value in vehicle group both heart rate and blood pressure during experimental period. The current study shows acute oral supplementation affected blood pressure or flow by gastric reflux, however elevation induced by flavan-3-ols lasted a number of minutes, and there were significant differences between distilled water and flavan-3-ols treatment rats. Further studies required to elucidate the mechanisms of initial rise in blood pressure and heart rate induced by flavan-3-ols. It is well known that eNOS phosphorylation can be induced via shear stress sensors in the plasma membrane of endothelial cells [22], [23]. Increased shear stress was reported to induce nitric oxide synthase (eNOS) dissociation from caveolin and binding of calmodulin and hsp90 complex. Caveolin-free eNOS undergoes various protein kinase-mediated phosphorylation and changes to an active form, finally catalyzing the conversion of molecular oxygen to nitric oxide (NO) using a terminal guanidino group from L-alginine [24]. In the present study, aortic eNOS was significantly phosphorylated 60 min after treatment with flavan-3-ols, resulting in an elevation of nitrate and nitrite concentrations in blood (Fig.4). In previous reports, acute physiological stress caused a rise in blood pressure and local blood flow that caused vasodilatation through induction of endothelial-derived relaxing factors [16], [17]. In addition, interventional trials have indicated a significant elevation of FMD several hours after chocolate ingestion. A chocolate-induced FMD has been estimated by the rise in NO production, since there was elevation of nitrate and nitrite levels in blood [13]–[15]. The results of the present study suggested that the elevation of blood flow in skeletal muscle by treatment with flavan-3-ols was mediated by increased NO production in endothelial cells. These hemodynamic changes resulted in increased shear stress, which led to more NO release and vasodilatation. The NO-induced vasodilatation probably acted to reduce the elevated blood pressure [25], [26].

Several previous studies was indicated that (-)-epicatechin in flavan-3-ols activated eNOS directly [27]–[29]. Although it was well investigated that (-)-epicatechin was distributed in blood as metabolites as glucuronide and/or sulfate forms [30], [31]. In addition, there was few information regarding the effect epicatechin oligomers on eNOS activities [32]. It was also known that epicatechin oligomers were poorly absorbed in blood [33], [34]. The contribution of the component in flavan-3-ols to eNOS activation and its mechanisms may be required additional discussion.

In this study, we found an increase in newly recruited capillaries in the cremaster muscle (Fig. 2b), and the elevation of blood flow in cremasteric artery was continued after blood pressure was returned to normal level. It is well established that an increased number of functioning capillaries is detected in tissues with a decreased oxygen partial pressure [35]. For example, the number of newly recruited capillaries is increased by contraction of skeletal muscle, which requires O_2_ for ATP production during exercise [36]. Further investigation is needed to elucidate the mechanism for increased capillary recruitment in the cremaster muscle by the ingestion of flavan-3-ols.

### Conclusions

In conclusion, we found an increase in RBC velocity in arterioles and blood flow in artery in the cremaster muscle, along with transient elevation of blood pressure and heart rate after a single oral administration of flavan-3-ols. In this condition, the elevation of shear stress on endothelial cells was confirmed by microscopic observation. These results suggest that the mechanism of FMD elevation after acute chocolate administration was partly due to enhanced NO release from endothelial cells due to increased shear stress.

## Supporting Information

Video S1 **Microcirculation in cremaster muscle of before administration of flavan-3-ols.**
(MPG)

Video S2 **Microcirculation in cremaster muscle of 5 min after administration of flavan-3-ols.**
(MPG)

## References

[1] Hammerstone JF, Lazarus SA, Mitchell AE, Rucker R, Schmitz HH (1999) Identification of procyanidins in cocoa (Theobroma cacao) and chocolate using high-performance liquid chromatography/mass spectrometry. J Agric Food Chem 47: 490–496. 10563922 10.1021/jf980760h

[2] Hatano T, Miyatake H, Natsume M, Osakabe N, Takizawa T, et al (2002) Proanthocyanidin glycosides and related polyphenols from cacao liquor and their antioxidant effects. Phytochemistry 59: 749–758. 11909632 10.1016/s0031-9422(02)00051-1

[3] Sanbongi C, Osakabe N, Natsume M, Takizawa T, Gomi S, et al (1998) Antioxidative polyphenols isolated from *Theobroma* cacao. J Agric Food Chem 46: 454–457. 10554262 10.1021/jf970575o

[4] Buitrago-Lopez A, Sanderson J, Johnson L, Warnakula S, Wood A, et al(2011) Chocolate consumption and cardiometabolic disorders: systematic review and meta-analysis. Available: http://www.bmj.com/content/343/bmj.d4488 Accessed 5 December 2013. 10.1136/bmj.d4488 PMC3163382 21875885

[5] Larsson SC, Virtamo J, Wolk A (2012) Chocolate consumption and risk of stroke: a prospective cohort of men and meta-analysis. Neurology 79: 1223–1229. 22933736 10.1212/WNL.0b013e31826aacfa

[6] Taubert D, Roesen R, Schömig E (2007) Effect of cocoa and tea intake on blood pressure: a meta-analysis. Arch Intern Med 167: 626–634. 17420419 10.1001/archinte.167.7.626

[7] Hooper L, Kroon PA, Rimm EB, Cohn JS, Harvey I, et al (2008) Flavonoids, flavonoid-rich foods, and cardiovascular risk: a meta-analysis of randomized controlled trials. Am J Clin Nutr 88: 38–50. 18614722 10.1093/ajcn/88.1.38

[8] Desch S, Schmidt J, Kobler D, Sonnabend M, Eitel I, et al (2010) Effect of cocoa products on blood pressure: systematic review and meta-analysis. Am J Hypertens 23: 97–103. 19910929 10.1038/ajh.2009.213

[9] Ried K, Sullivan T, Fakler P, Frank OR, Stocks NP. (2010) Does chocolate reduce blood pressure? A meta-analysis.BMC Med. 28;8:39 Available: http://www.biomedcentral.com/1741-7015/8/39. Accessed 5 December 2013. 10.1186/1741-7015-8-39 PMC2908554 20584271

[10] Tokede OA, Gaziano JM, Djoussé L (2011) Effects of cocoa products/dark chocolate on serum lipids: a meta-analysis. Eur J Clin Nutr 65: 879–886. 21559039 10.1038/ejcn.2011.64

[11] Shrime MG, Bauer SR, McDonald AC, Chowdhury NH, Coltart CE, et al (2011) Flavonoid-rich cocoa consumption affects multiple cardiovascular risk factors in a meta-analysis of short-term studies. J Nutr 141: 1982–1988. 21956956 10.3945/jn.111.145482

[12] Hooper L, Kay C, Abdelhamid A, Kroon PA, Cohn JS, et al (2012) Effects of chocolate, cocoa, and flavan-3-ols on cardiovascular health: a systematic review and meta-analysis of randomized trials. Am J Clin Nutr 95: 740–751. 22301923 10.3945/ajcn.111.023457

[13] Schroeter H, Heiss C, Balzer J, Kleinbongard P, Keen CL, et al (2006) (-)-Epicatechin mediates beneficial effects of flavanol-rich cocoa on vascular function in humans. Proc Natl Acad Sci U S A 103: 1024–1029. 16418281 10.1073/pnas.0510168103 PMC1327732

[14] Monahan KD, Feehan RP, Kunselman AR, Preston AG, Miller DL, et al (1985) Dose-dependent increases in flow-mediated dilation following acute cocoa ingestion in healthy older adults. J Appl Physiol 111: 1568–1574. 10.1152/japplphysiol.00865.2011 PMC3233882 21903881

[15] Balzer J, Rassaf T, Heiss C, Kleinbongard P, Lauer T, et al (2008) Sustained benefits in vascular function through flavanol-containing cocoa in medicated diabetic patients a double-masked, randomized, controlled trial. J Am Coll Cardiol 51: 2141–2149. 18510961 10.1016/j.jacc.2008.01.059

[16] Birk GK, Dawson EA, Batterham AM, Atkinson G, Cable T, et al (2013) Effects of exercise intensity on flow mediated dilation in healthy humans. Int J Sports Med 34: 409–414. 23041960 10.1055/s-0032-1323829

[17] Katayama K, Fujita O, Iemitsu M, Kawano H, Iwamoto E, et al (2013) The effect of acute exercise in hypoxia on flow-mediated vasodilation. Eur J Appl Physiol 113: 349–357. 22729610 10.1007/s00421-012-2442-5

[18] Chen H, Zhang L, Zhang M, Song X, Zhang H, et al (2013) Relationship of depression, stress and endothelial function in stable angina patients. Physiol Behav 118: 152–158. 23688945 10.1016/j.physbeh.2013.05.024

[19] Osakabe N, Yamagishi M, Sanbongi C, Natsume M, Takizawa T, et al (1998) The antioxidative substances in cacao liquor. J Nutr Sci Vitaminol 44: 313–321. 9675711 10.3177/jnsv.44.313

[20] Natsume M, Osakabe N, Yamagishi M, Takizawa T, Nakamura T, et al (2000) Analyses of polyphenols in cacao liquor, cocoa, and chocolate by normal-phase and reversed-phase HPLC. Biosci Biotechnol Biochem 64: 2581–2587. 11210120 10.1271/bbb.64.2581

[21] Misko TP, Schilling RJ, Salvemini D, Moore WM, Currie MG (1993) A fluorometric assay for the measurement of nitrite in biological samples. Anal Biochem 214: 11–16. 7504409 10.1006/abio.1993.1449

[22] Wentzel JJ, Chatzizisis YS, Gijsen FJ, Giannoglou GD, Feldman CL, et al (2012) Endothelial shear stress in the evolution of coronary atherosclerotic plaque and vascular remodelling: current understanding and remaining questions. Cardiovasc Res 96: 234–243. 22752349 10.1093/cvr/cvs217

[23] Yamamoto K, Ando J (2011) New molecular mechanisms for cardiovascular disease:blood flow sensing mechanism in vascular endothelial cells. J Pharmacol Sci 116: 323–331. 21757846 10.1254/jphs.10r29fm

[24] Davis ME, Cai H, Drummond GR, Harrison DG (2001) Shear stress regulates endothelial nitric oxide synthase expression through c-Src by divergent signaling pathways. Circ Res 89: 1073–1080. 11717166 10.1161/hh2301.100806

[25] Rakobowchuk M, Harris E, Taylor A, Baliga V, Cubbon RM, et al (2012) Heavy and moderate interval exercise training alters low-flow-mediated constriction but does not increase circulating progenitor cells in healthy humans. Exp Physiol. 97: 375–385. 22179420 10.1113/expphysiol.2011.062836 PMC3505374

[26] Green DJ, Maiorana A, O'Driscoll G, Taylor R (2004) Effect of exercise training on endothelium-derived nitric oxide function in humans. J Physiol 561: 1–25. 15375191 10.1113/jphysiol.2004.068197 PMC1665322

[27] Ramirez-Sanchez I, Maya L, Ceballos G, Villarreal F (2010) (-)-epicatechin activation of endothelial cell endothelial nitric oxide synthase, nitric oxide, and related signaling pathways. Hypertension 55: 1398–1405. 20404222 10.1161/HYPERTENSIONAHA.109.147892 PMC2874202

[28] Ramirez-Sanchez I, Maya L, Ceballos G, Villarreal F (2011) (-)-Epicatechin induces calcium and translocation independent eNOS activation in arterial endothelial cells. Am J Physiol Cell Physiol 300: C880–C887. 21209365 10.1152/ajpcell.00406.2010 PMC3074631

[29] Brossette T, Hundsdörfer C, Kröncke KD, Sies H, Stahl W (2011) Direct evidence that (-)-epicatechin increases nitric oxide levels in human endothelial cells. Eur J Nutr 50: 595–599. 21327831 10.1007/s00394-011-0172-9

[30] Baba S, Osakabe N, Natsume M, Muto Y, Takizawa T, et al (2001) *In vivo* comparison of the bioavailability of (+)-catechin, (−)-epicatechin and their mixture in orally administered rats. J Nutr 131: 2885–2891. 11694613 10.1093/jn/131.11.2885

[31] Natsume M, Osakabe N, Oyama M, Sasaki M, Baba S, et al (2003) Structures of (-)-epicatechin glucuronide identified from plasma and urine after oral ingestion of (-)-epicatechin: differences between human and rat. Free Radic Biol Med 34: 840–849. 12654472 10.1016/s0891-5849(02)01434-x

[32] Byun EB, Ishikawa T, Suyama A, Kono M, Nakashima S, et al (2012) A procyanidin trimer, C1, promotes NO production in rat aortic endothelial cells via both hyperpolarization and PI3K/Akt pathways. Eur J Pharmacol 692: 52–60. 22796647 10.1016/j.ejphar.2012.07.011

[33] Baba S, Osakabe N, Natsume M, Terao J (2002) Absorption and urinary excretion of procyanidin B2 [epicatechin-(4beta-8)-epicatechin] in rats. Free Radic Biol Med 33: 142–148. 12086692 10.1016/s0891-5849(02)00871-7

[34] Spencer JP, Schroeter H, Rechner AR, Rice-Evans C (2001) Bioavailability of flavan-3-ols and procyanidins: gastrointestinal tract influences and their relevance to bioactive forms *in vivo* . Antioxid Redox Signal 3: 1023–39. 11813978 10.1089/152308601317203558

[35] Webb RC, Myers JH (1979) Skeletal muscle capillary densities during reactive hyperemia. Experientia 35: 1476–1477. 510489 10.1007/BF01962794

[36] Honig CR, Odoroff CL, Frierson JL (1980) Capillary recruitment in exercise: rate, extent, uniformity, and relation to blood flow. Am J Physiol 238: H31–H42. 7356032 10.1152/ajpheart.1980.238.1.H31

